# Exploiting Current Understanding of Hypoxia Mediated Tumour Progression for Nanotherapeutic Development

**DOI:** 10.3390/cancers11121989

**Published:** 2019-12-11

**Authors:** Jie Feng, Niall M. Byrne, Wafa Al Jamal, Jonathan A. Coulter

**Affiliations:** School of Pharmacy, Queens University Belfast, Lisburn Road, Belfast BT9 7BL, UK; jfeng09@qub.ac.uk (J.F.); n.byrne@qub.ac.uk (N.M.B.); w.al-jamal@qub.ac.uk (W.A.J.)

**Keywords:** angiogenesis, epithelial-to-mesenchymal transition, hypoxia, immunosuppression, metabolism, nanoparticle, nanotherapeutics, tumour microenvironment

## Abstract

Hypoxia is one of the most common phenotypes of malignant tumours. Hypoxia leads to the increased activity of hypoxia-inducible factors (HIFs), which regulate the expression of genes controlling a raft of pro-tumour phenotypes. These include maintenance of the cancer stem cell compartment, epithelial-mesenchymal transition (EMT), angiogenesis, immunosuppression, and metabolic reprogramming. Hypoxia can also contribute to the tumour progression in a HIF-independent manner via the activation of a complex signalling network pathway, including JAK-STAT, RhoA/ROCK, NF-κB and PI3/AKT. Recent studies suggest that nanotherapeutics offer a unique opportunity to target the hypoxic microenvironment, enhancing the therapeutic window of conventional therapeutics. In this review, we summarise recent advances in understanding the impact of hypoxia on tumour progression, while outlining possible nanotherapeutic approaches for overcoming hypoxia-mediated resistance.

## 1. Introduction

The hostile microenvironment within a solid tumour is increasingly recognized as a major impediment to effective cancer therapy [1]. Hypoxia, a hallmark of malignancy, is one of the most typical and important features of the tumour microenvironment (TME), caused by the imbalance between oxygen supply and consumption by cancer and stromal cells [2,3]. Failure of the local environment to overcome this deficit due to the aberrant vascular architecture results in tumour hypoxia. Hypoxia has been shown to contribute to malignant progression and treatment failure, in particular, resistance to radiotherapy.

### 1.1. Defining Tumour Hypoxia

Since the development of the oxygen electrode, direct measurements of tissue oxygenation has revealed considerable heterogeneity in oxygen concentration in normal and pathological tissue. Physiological hypoxia is typically defined as ≤2% O_2_ (15 mmHg), while pathological hypoxia defined as ≤1% O_2_ and radiobiological hypoxia as ≤0.4% [3]. Hypoxia is classified as perfusion-limited (acute) hypoxia or diffusion-limited (chronic) hypoxia [4]. Perfusion-limited hypoxia is often caused by the structural and functional abnormality of tumour microvasculature, characterized by an immature endothelial cell lining and basement membrane, disorganized vascular network and wide intercellular spaces. These structural abnormalities lead to the rapid oxygen fluctuations between hypoxia, anoxia and reoxygenation [4]. The lifetime of perfusion related hypoxia ranges from less than a minute to several hours in experimental tumours [5]. In contrast, diffusion-limited hypoxia is mainly due to an increase in diffusion distance, attributed to a rapidly expanding tumour. Tumour cells are often far from nutritive blood vessels, where most of the accessible molecular oxygen is consumed by proliferating cells before diffusion to deep tumour layers occurs. This results in the development of a hypoxic tumour core [6].These two forms of tumour hypoxia often overlap spatio-temporally, influencing the interaction between cancer, stromal and immune host cells. Additionally, tissue oxygenation may also be perturbed by anaemia, which can often occur following chemotherapy, radiotherapy, blood loss and low haemoglobin levels [7].

### 1.2. Implications of Tumour Hypoxia and Nanotherapeutic Opportunities

It has previously been suggested that up to 60% of solid tumours contain hypoxic or anoxic regions, conferring major implications for chemo- and radiotherapy [8]. Biologically, hypoxia can trigger proteomic alterations within neoplastic and stromal cells, further promoting malignant progression and poor survival. Furthermore, hypoxia is the leading cause of treatment failure for radiotherapy and photodynamic therapy since both approaches rely on the creation of reactive oxygen species. For chemotherapy, solid tumour hypoxia is associated with elevated HIF gene expression, promoting double-strand DNA repair and subsequently, chemo-resistance [9]. Hypoxia is also a potential barrier to immunotherapy. Several studies suggest that the recruitment of immunosuppressive cells within hypoxic regions promote immune suppression. Furthermore, hypoxia-driven adaptive mechanisms diminish the immune cell response via expression of immune check-point molecules such as PDL-1 (programmed death ligand-1) and HLA-G (human leukocyte antigen G), altering both tumour metabolism and metabolite formation [10]. Nanotherapeutics offer a unique approach to exploit the physiological and pathophysiological response to hypoxia within the TME. Interest in the use of nanoparticles (NPs) for biological applications, including enhanced drug delivery, diagnostic imaging and as radiosensitisers, has increased over the last 25 years [11,12].

### 1.3. Scope of the Review

**Figure 1:**
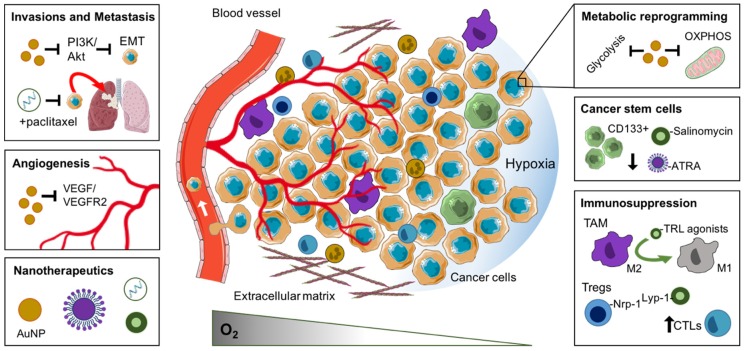
Nanotherapeutic approaches to exploit the hypoxic tumour microenvironment. The response of the tumour microenvironment to reduced oxygenation, including cancer stem cell enrichment, angiogenesis, invasion and metastasis, metabolic reprogramming and immunosuppression, and the nanotherapeutic approaches to exploit or manipulate these features. Abbreviations: AuNP, gold nanoparticle; CTL, cytotoxic T lymphocyte; EMT, epithelial to mesenchymal transition; MDSCs, myeloid-derived suppressor cells; OXPHOS, oxidative phosphorylation; TAM, tumour-associated macrophage.

In this review, we summarise recent advances relating to the biological consequence and therapeutic efficacy of tumour hypoxia [13,14]. We outline the negative impact of tumour hypoxia on the propagation of cancer stem cells, malignant progression, metastasis immunosuppression and metabolic reprogramming. We also consider the use of nanoparticles to manipulate hypoxia-induced features of the TME for therapeutic gain (Figure 1).

## 2. Biological Response and Therapeutic Opportunities of Tumour Hypoxia

**Figure 2:**
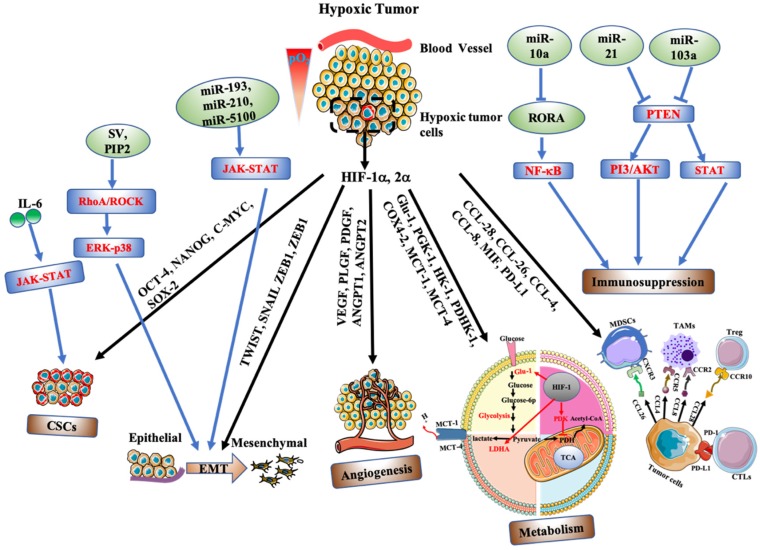
Hypoxia regulates tumour progression via various mechanisms, including HIF independent (blue arrow) and HIF-dependent manner (black arrow). Intratumoural hypoxia causes overexpression of HIF-1α and HIF-2α, leading to transactivation of multiple target genes outlined above. These regulate the cellular response to CSCs maintenance, EMT induction, angiogenesis, metabolic reprogramming, and immunosuppression. Tumour progression is also influenced by numerous HIF-independent factors including miRNA expression and cytokine release, activating various pro-tumour signalling pathways. Abbreviations: IL-6, interleukin 6; SV, supervillain; PIP2, phosphatidylinositol (4,5) biphosphate; RORA, RAR-related orphan receptor; MDSCs, myeloid-derived suppressor cells; TAMs, tumour-associated macrophages; Treg, regulatory T cells; CTLs, cytotoxic T lymphocytes.

The presence of hypoxia strongly correlates with an aggressive tumour phenotype, therapeutic resistance and poor patient survival. Initially, we outline the mechanisms by which hypoxia promotes an aggressive tumour phenotype, and discuss the treatment opportunities that may exist using nanotherapeutic strategies (Table 1). The full biological response and therapeutic implications to hypoxia are extensive and well beyond the limits of this review paper. As such, we will review only recent discoveries related to the cellular response of hypoxia-driven malignant progression and resistance, which have been summarised in Figure 2.

### 2.1. Enrichment and Propagation of Cancer Stem Cells

Cancer stem cells (CSCs) or tumour initiating cells are a small subpopulation of cells which share progenitor-like characteristics including self-renewal, tumour initiation and multi-lineage differentiation. Furthermore, tumour hypoxia has been directly correlated with metastatic potential and treatment resistance. CSCs can induce cell cycle arrest, conferring resistance to both chemo- and radiotherapy. Post-treatment, surviving CSCs are released from dormancy driving repopulation and dissemination. Therefore, a deep understanding of the influence of hypoxia in CSCs biology is central to the development of future therapeutic approaches.

The CSCs model is driven by many key regulatory factors including genetic diversity, epigenetics and the TME. Recent evidence points to the influence of the microenvironment on cell plasticity and differentiation. Tumour hypoxia is a key environmental stress associated with CSC self-renewal, epithelial-to-mesenchymal transition (EMT) and treatment resistance. Hypoxia expands the CSCs population through several molecular mechanisms. Hypoxia mediated epigenetics act as a possible driving force for promoting cancer stemness. These epigenetic factors include DNA methylation, histone modification, chromatin remodelling and microRNA expression. Kang et al. (2019) identified hypoxia as a key driver of cancer stemness and EMT in multiple lung cancer models, driven by a decrease in E-cadherin and a corresponding increase in the mesenchymal markers fibronectin, vimentin, α-SMA, slug and ZEB1 [50]. Additionally, significant activation of the CSC marker CXCR4 was reported. The impact of CXCR4 was demonstrated following the induction of strong CXCR4 immunoreactivity following the intratumoural injection of hypoxic cells. Furthermore, methylation-specific PCR and sequencing data further confirmed a decrease in CXCR4 promoter methylation under hypoxia, thus promoting CXCR4 expression and the acquisition of CSC-like properties. Prasad et al. (2017) investigated the role of hypoxia in regulating stemness in an aggressive glioblastoma tumour model [51]. The authors reported hypoxia-mediated self-renewal of A172 cells through elevated neurosphere formation. Furthermore, at the molecular level, OCT-4, NANOG, SOX-2 and Stat3 gene and protein expression were highly upregulated following chronic hypoxia (Figure 2). Importantly, hypoxia was shown to reduce 5-methyltosine (5-mC) expression by at least three-fold at all OCT-4 regulatory regions (OA, OB, OC), and within the promoter region of NANOG. Furthermore, a concomitant enrichment of 5-hydroxymethylcytosine (5-hmC) at OCT-4 regions (OA (2.5-fold), OB (4.6-fold)), together with a significant reduction of H3K27me3 methylation confirmed hypoxia-meditated regulation of stem associated genes. N6-methyladenosine (m6A) modification has recently been identified as an important regulator of stem cell pluripotency. Zhang et al. (2016) showed that hypoxia induced HIF-1α and HIF-2α dependent expression of AlkB homolog 5 (ALKBH5) [52], a m6A demethylase in breast cancer models. This resulted in demethylation at m6A residues within the 3′-UTR of NANOG, upregulating functional NANOG protein and contributing to breast CSC enrichment.

The influence of hypoxia on cell cycle progression in glioma CSCs has been demonstrated by Li et al. (2013) [53]. Cells maintained in 1% O_2_ for 48 h significantly increased G_0_/G_1_ accumulation with a corresponding reduction in G_2_/M cells, indicating elevated quiescence. This phenotype corresponded with elevated OCT-4 and SOX-2 and reduced expression of GFAP, a marker of stem cell differentiation, implying that hypoxia stemness is primarily attributed to dedifferentiation. Hypoxia has also been shown to promote dedifferentiation of mature glioma cells into stem-like glioma stem cells (GSCs). Hypoxia induced single differentiated CD133-/CD15-/NESTIN- glioma cells into viable neurospheres through elevated expression of critical genes including SOX-2, OCT-4, KLF-4, NANOG, CD133, CD15, NESTIN and ABCG2 [54]. Interestingly, hypoxia induced CSC enrichment also resulted in increased tumourgenicity and mortality in vivo. At the molecular level, higher levels of HIF-1α were measured in both neurosphere and tumour samples, with the importance of HIF-1α further illustrated through interference experiments, potently suppressing neurosphere formation and stem cell marker expression (CD133, CD15 and NESTIN).

Recent mechanistic evidence supported the CSC maintenance function of hypoxia stimulated JAK-STAT signalling in breast cancer models [55]. Conditioned medium (CM) from hypoxic estrogen receptor (ER-α) positive tumour cells enriched the fraction of CSCs compared to normal growth conditions. Conversely, conditioned medium from ERα -negative tumour cells decreases the CSC subpopulations. The authors reported that JAK-STAT signalling activity regulates the contrasting secretome of ERα positive and ERα negative breast cancer cells, through differential cytokine (IL6, IL12RB2) secretion dependent on estrogen receptor status, acting as a key regulator of JAK-STAT phosphorylation (Figure 2).

#### Nanotherapeutic Approaches to Target Cancer Stem Cells

Targeting CSCs is of particular interest, given their purported therapeutic resistance and capacity for tumour repopulation following treatment. ALDH, CD44, CD133 and other cell surface markers outlined above have frequently been used as putative CSC markers. In addition, CSCs are dependent on cell signalling pathways including Wnt and Notch, which may act as therapeutic drug targets for intervention [56,57]. A number of therapeutic agents that have effects on eliminating or inhibiting CSCs have been proposed and confirmed, including, doxorubicin, paclitaxel, salinomycin, curcumin and all-trans retinoic acid. However, most of the agents have characteristics limiting their effective application in vivo, including poor solubility, low specificity, poor stability, and short circulation time. Nanotechnology drug delivery approaches hold significant potential for tackling these limitations. A series of therapeutic agents have been loaded into CD133 or CD44 functionalized nanosystems, demonstrating an increased efficacy for eliminating CSC populations both in vitro and in vivo. Doxorubicin, a widely used clinical agent, was loaded into chitosan decorated NPs, with the nanoformulation exhibiting six-fold increased cytotoxicity over free doxorubicin. Importantly, this approach was shown to eliminate CD44+ CSCs-like cells leading to a significant reduction in tumour growth [20].

All-trans retinoic acid (ATRA), an active metabolite of vitamin A, has shown therapeutic efficacy in modulating CSC subpopulations, evidenced by reductions in the CSC markers CD44 and ALDH in gastric carcinoma models in vitro and in vivo and the attenuation of CSC-like properties in ALDH-high expressing ovarian CSCs [58,59]. When ATRA was incorporated into lipid-polymer NPs conjugated with CD133 aptamers, increased targeting and therapeutic efficacy against osteosarcoma CSCs was reported over ATRA alone (tumour volume inhibitory rates of 81.1% and 44.3%, respectively) [18]. Salinomycin, a polyether ionophore antibiotic, has also shown potential in killing CSCs. Poly (lactic-co-glycolic acid) NPs conjugated with CD133 aptamers, specifically delivered salinomycin to CD133+ Saos-2 CSCs significantly attenuating osteosarcoma tumour growth in comparison to salinomycin-only treatment (7.1-fold increase in tumour growth over 60 days in comparison to 17.4-fold increase) as well as reducing the frequency of CD133+ cells in vivo [15]. Similar observations have been shown in models of ovarian cancer in vivo, and in models of osteosarcoma following dual targeting with CD133 and EGFR aptamers [16,17]. Curcumin, a well-known dietary polyphenol derived from the rhizomes of turmeric, has shown excellent therapeutic efficacy against CSCs through the suppression of both CSC self-renewal pathways (Wnt/β-catenin, hedgehog (Hh), and Notch) and specific microRNA involved in the acquisition of EMT [60]. Furthermore, curcumin loaded CD44+ targeting nanomicelles resulted in the potent suppression of pro-tumour NF-κB signalling [19]. The inhibition of Notch signalling using γ-secretase inhibitors has been shown to slow tumour growth using xenograft models of medulloblastoma and by reducing CSC (CD133+) subpopulations [61]. However, inhibiting key CSC signalling pathways may inadvertently affect normal stem cell function. Targeted inhibition of Notch signalling in breast CSCs was achieved using a γ-secretase inhibitor loaded on a glucose-functionalised mesoporous silica nanoparticle. These co-functionalised NPs successfully reduced the MDA-MB-231 CSC ALDH side-population in a chick embryo chorioallantoic membrane (CAM) model, reducing tumour growth in vivo [26].

The combination of chemotherapy agents and salinomycin or ATRA has also received increased attention due to an enhanced synergy achieved through eradicating both terminally differentiated tumour cells and CSCs (Figure 1). For example, Gong et al. (2016) reported that a nanoliposome delivery system co-delivering salinomycin and doxorubicin possessed the best tumour inhibitory rate, significantly reducing the percentage of liver CSCs in vivo [21]. A similar study reported that co-delivery of salinomycin and paclitaxel using hyaluronic acid decorated poly (lactic-co-glycolic acid) NPs showed the highest cytotoxicity against CD44+ breast CSCs compared to salinomycin or paclitaxel used as monotherapies [22]. Sun et al. (2015) also reported that co-delivery of ATRA and doxorubicin in a nanoparticle formulation effectively delivered the agents to both non-CSCs and CSCs, forcing CSC differentiation into a more treatment sensitive phenotype, with the effect of markedly suppressing tumour growth [23].

Recently, ATP-binding cassette subfamily G member 2 (ABCG2), a member of the ABC transporter family, has also been recognized as a promising target for CSCs. ABCG2 has been proposed as the main driver contributing to a subpopulation of slow-cycling CSCs, endowed with enhanced tumourigenic potential and multidrug resistance [62]. Targeting and suppressing ABCG2 function, therefore, represents a sensible strategy to sensitise CSCs populations to chemotherapy. For example, Qi et al. (2015) loaded NPs with siRNA targeting ABCG2 and a chemotherapeutic (cisplatin, 5-fluoroucail or paclitaxel) for the treatment of CD133+ laryngeal carcinoma. The authors reported that the downregulation of ABCG2 significantly enhanced chemotherapeutic drug-induced apoptosis, leading to superior control of tumour growth [24]. Similarly, co-delivery of wedelolatone (Wdl) and paclitaxel incorporated within PLGA NPs downregulated the ABCG2 and SOX-2 expression, sensitising tumour cells to paclitaxel treatment, and reducing the overall percentage of ALDH+ CSCs in vitro and in solid tumours [25].

### 2.2. Invasions and Metastasis

Malignant tumours frequently exhibit hypoxia and nutrient deprivation, closely correlated with therapeutic treatment resistance and tumour relapse. Despite significant advances in the treatment of metastatic disease, the underlying mechanisms of metastasis are less well developed. Growing evidence indicates that the hypoxic microenvironment promotes tumour progression by triggering a series of transcriptional responses that regulate migration, invasion, cell proliferation, angiogenesis and cell metabolism, ultimately contributing to an aggressive tumour phenotype [63,64].

Metastasis is a complicated process comprised of a series of highly regulated steps where tumour cells gain more invasive properties. It begins with a change in tumour plasticity, through a process called epithelial-mesenchymal transition (EMT), where epithelial cells lose cell–cell adherence, endowing tumour cells with an enhanced migratory and invasive potential [65]. A critical hallmark of EMT is the repression of E-cadherin expression and the upregulation of associated mesenchymal genes. This transformation results in the disruption of cell–cell adhesion and cell polarity. Hypoxia can induce EMT and invasion via the regulation of EMT-associated transcriptional factors including TWIST, SNAIL, ZEB1, ZEB2 (Figure 2) [66,67]. Pancreatic cancer cells display enhanced cell proliferation and EMT under hypoxic conditions, mediated through an upregulation of HIF-1α and TWIST, corresponding with a dramatic decrease in the expression of E-cadherin and p16Ink4A (p16). However, knockdown of HIF-1α was shown to mask the effect of TWIST overexpression, hypoxia-induced EMT and proliferation through HIF-1α /TWIST signalling, indicating the dominant nature of HIF-1α expression [68].

EMT induction is also controlled by other regulatory mechanisms, with recent studies reporting the influence of specific miRNAs. MicroRNAs suppress protein expression through a combination of mRNA destabilisation and translational repression. Recent reports have shown that hypoxia alters miRNA expression including exosome derived miR-193a-3p, miR-210-3p and miR-5100 (released from hypoxic bone marrow-derived mesenchymal stem cells), promoting epithelial cancer cell invasion and lung metastasis through JAK-STAT overactivation (Figure 2) [69]. Similarly, hypoxia was shown to upregulate miR-210-5P and miR-210-3p [70], upstream precursors N-cadherin, Twist, MMP-2 in hepatoma cell models. Inhibition of miR-210-5P and miR-210-3p suppressed EMT induction and cell progression, indicating that hypoxia-induced miR-210-5p and miR-210 3p are important regulators of a hypoxia-induced metastatic phenotype. Hypoxia-regulated expression of miR-310a-3p has also been shown to push macrophage differentiation towards an M2 phenotype in a HIF-1α or HIF-2α dependent manner [71]. Importantly, invasion and migration potential appeared to be significantly enhanced in macrophages exposed to miR-301a-3p loaded exosomes, providing a mechanism by which hypoxia can stimulate immune cell-mediated tumour progression.

Exosome liberated miR-310a-30p is understood to polarize macrophages into an M2 phenotype via the activation of PTEN/P13Kγ signalling, favouring the malignant properties of tumour cells, due in part to the expression of the anti-inflammatory cytokine (IL-10) and arginase-1 (Arg1) [72]. Additionally, hypoxia can stimulate EMT through a number of other cell-signalling pathways which include RhoA/ROCK-ERK/P38, PI3/Akt. Kaneka et al. (2016) showed that hypoxia treatment induced cell invasion, migration and EMT using oral squamous cell carcinoma (OSCC) cell lines [73]. The authors reported that overexpression of HIF-1α triggers PI3/Akt signalling and the subsequent phosphorylation of GSK3-β (p-GSK3-β). Conversely, inhibition of PI3/Akt signalling led to reduced phosphorylation of GSK3-β and Akt, suppressing EMT induction. These data suggest that hypoxia-induced pGSK3-β is an important regulator in the invasion and metastasis of OSCC. Additionally, supervillin (SV), a protein with two recognised isoforms (SV4 and SV5) was also shown to be upregulated in HCC tumour models under hypoxic conditions, with elevated SV4 and SV5 levels associated with enhanced cell migration and reorganization of the action cytoskeleton, thus promoting EMT [74]. RhoA/Rock and MAPK/ERK/p38 signalling were identified as companion proteins involved in supervillin-driven HCC migration and invasion. Perhaps more importantly, ERK/p38 phosphorylation is downstream of RhoA/ROCK activation. Therefore, it could be suggested that supervillain-induced EMT is mainly mediated through the RhoA/ROCK and ERK/p38 pathways. Similar results were reported by Huang et al. (2019) in hepatocellular carcinoma models, where the induction of EMT due to actin cytoskeleton remodelling was controlled by the negative regulation of CAPZA1 (capping actin protein of muscle Z-line alpha subunit 1; Figure 2) [75]. As such, the downregulation of CAPZA1 promoted cell invasion, migration and the induction of EMT. CAPZA1 regulation in actin remodelling was primarily mediated via the interaction between CAPZA1 and phosphatidylinositol (4,5) bisphosphate (PIP2), in which the combination of PIP2 and CAPZA1 would lead to CAPZA1 depletion and a subsequent increase in F-actin levels. The authors reported that levels of PIP2 under hypoxia could be modulated by HIF-1α/RhoA/Rock1 signalling, in which hypoxia treatment led to the increased expression of HIF-1α, RhoA and Rock1, hence resulting in elevated PIP2 levels and subsequent actin cytoskeleton remodelling.

#### Targeting EMT and Metastatic Progression with Nanoparticle Formulations

As outlined above, the ability of cancer cells to invade local tissue and spread to distant sites is a critical step in disease progression, often accompanied by a poorer clinical prognosis. EMT is the proposed mechanism by which cells acquire the properties necessary for invasion and migration. Intrinsic properties of NPs have the potential to inhibit cancer progression through the regulation of EMT. For example, Arvizao et al. (2013) reported that gold NP (AuNP) treatment can delay tumour metastases through the inhibition of MAPK signalling and EMT reversal. They found that unmodified AuNPs not only downregulated the phosphorylation of MAPK but also reversed EMT by downregulating Snail, N-Cadherin, Vimentin [27]. Polyethylenimine coated superparamagnetic iron NPs (SPIONs) are proven to inhibit tumour cell migration and invasion through the inhibition of Src kinase activity and downregulation of MT1-MMP and MMP2 matrix-metalloproteinases. In addition, SPIONs treatment can downregulate miR-21, upregulating cell migration inhibitors PTEN, PDCD4 and sproutyl-1 [28].

Targeting metastatic signalling pathways involving EMT induction represents another approach for inhibiting metastasis [76]. PEGylated AuNPs combined with cold plasma, were proven to inhibit glioblastoma cell proliferation in vitro by blocking PI3K/Akt signalling (Figure 1). In addition, co-treatment of glioma xenografts suppressed tumour growth and mesenchymal markers expression including N-Cad, Zeb-1 and Slug, while increasing the epithelial cell marker E-cadherin; suggesting a reversal of EMT [31]. The aberrant activation of JAK-STAT signalling confers malignant properties to cancer cells, including EMT induction and malignant progression [77]. Inhibition of JAK-STAT signalling has been shown to reduce cancer proliferation and metastasis. Guo et al. (2019) loaded miR-125-5p into a folate acid coated Fa-polyethyleneglycol (PEG)–g-polyetherimide (PEI) superparamagnetic iron oxide nanocarrier (SPIONs), evaluating its therapeutic effect against hepatocellular carcinoma (HCC). The authors reported that the miR-125-5p loaded nanomedicine effectively inhibited the EMT potential of HCC cells via the inhibition of STAT and the inactivation of Wnt/β-Catenin, inhibiting tumour growth in HCC-bearing mice [29]. Similar work by Huang et al. (2019) demonstrated that silica-coated zinc arsenite NPs (ZnAs@SiO_2_ NPs) significantly inhibited the proliferation, migration and invasion of HCC cell lines, attenuating in vivo tumour growth by 2.2-fold in comparison to NP-only control, mediated through the upregulation of SH2-containing protein tyrosine phosphatase 1 (SHP-1) and the corresponding suppression of JAK2/STAT3 signalling [30].

Hypoxia exposure leads to an increase in TWIST expression. Hyaluronic-acid conjugated mesoporous silica nanoparticles (MSN-HAs) loaded with TWIST siRNA successfully suppressed TWIST expression in vitro, subsequently reducing the tumour burden in a model of epithelial ovarian cancer in vivo [33]. Furthermore, amphiphilic polymer-based nanoparticles loaded with siRNAs against SNAIL and TWIST, and used in combination with the chemotherapeutic paclitaxel, inhibited tumour growth and metastasis of the 4T1 breast cancer model in vivo, while siRNA alone exhibited only modest benefits (Figure 1) [32]. Therefore, co-delivery of EMT targeted molecules loaded on nanoparticles may provide enhanced metastatic inhibition.

### 2.3. Angiogenesis

Abnormal angiogenesis is a common feature of tumour malignant progression, where rapid growing tumours outstrip oxygen supply, yielding a hypoxic tumour mass. Consequently, hypoxia induces the formation of new blood vessels in an attempt to ameliorate oxygen depletion stress. Hypoxia and HIF-1 expression trigger an imbalance between pro- and anti-angiogenic factors and cytokines modulating gene expression involved in the angiogenetic response [78,79]. This typically includes the activation of angiogenic genes and receptors such as VEGF (vascular endothelial growth factor), PLGF (placental growth factor), PDGFB (platelet-derived factor) and integrins among others (Figure 2) [80]. Integrins, particularly alpha v beta 3 (αvβ3), have been shown to be upregulated within the TME, expressed on both tumour cells and the vasculature [81]. Hypoxia (1%O_2_) has been shown to upregulate αv expression in human microvascular endothelial (HMEC-1) cells in vitro. Furthermore, knockdown of HIF-1α was shown to inhibit hypoxia stimulated β3-integrin expression, suggesting induction of β3-integrin is HIF-dependent [82]. The effect of HIF-1α on the angiogenic potential of small cell lung cancer (SCLC) significantly upregulated the expression of pro-angiogenic genes including VEGF-A, TNFA1P6, PDGFC, FN1, MMP 28 and MMP14 [83]. Myocyte enhancer factor 2D (MEF2D) has also been proven to play a central role in tumour angiogenesis [84]. MEF2D expression positively correlated with colorectal tumour angiogenesis, through the induction of pro-angiogenic factors including PDGF-BB, PDGF-C, PLGF, milk fat globule factor (MFG)-E8, and tumour necrosis factor superfamily member (TNFRSF). Furthermore, it was observed that MEF2D is a downstream effector of HIF-1α transcriptional activity.

As with EMT, exosomal miRNA has also been proven to promote angiogenesis. Matsuura et al. (2019) compared the angiogenic activity of HUVEC cells co-cultured with exosomes derived from hepatocarcinoma cells cultured under variable oxygen tensions [85]. Harvested exosomes collected under hypoxic stress displayed enhanced tubule formation in HUVECs cells, mediated through miR-155 upregulation. Conversely, miR-155 knockdown attenuated tubule formation, implying that exosomal miR-155 regulates angiogenic potential. Similarly, Hsu et al. (2017) demonstrated that exosomal miR-23a derived from lung cancer was significantly upregulated by hypoxia [86]. Exosomal miR-23a suppressed prolyl-hydroxylase 1 and 2 (PHD 1 and 2) and inhibited tight junction protein ZO-1, with the effect of increased vascular permeability and tumour cell transendothelial migration, effects reversed in knockout experiments.

#### Overcoming Hypoxia-Driven Angiogenesis Using Nanoparticles

Angiogenesis modulating strategies have largely focused on either inhibiting, disrupting or normalising the aberrant tumour vasculature. In this context, nanoparticles are at an advantage as they may exploit the leaky and aberrant vascular architecture of the TME, accumulating within tumour tissue via the enhanced permeability and retention (EPR) effect [87]. Interestingly, gold NPs (AuNPs) have been shown to have intrinsic anti-angiogenic effects in vivo, likely through inhibition of the VEGF/VEGFR2 signalling pathway (Figure 1) [34,88,89]. Evidence from an in vivo melanoma model has shown that AuNPs could normalise tumour vasculature, alleviate tumour hypoxia and reduce metastatic spread to the lungs (20% of AuNP-treated tumour-bearing animals developed metastasis in comparison to 66.7% of control-treated) [35]. However, the anti-angiogenic potential of AuNPs appears to be transient: In a xenograft model of colorectal cancer (CRC), AuNPs treatment reduced vessel density and increased pericyte coverage concomitant with vascular normalization and improvements in tumour hypoxia until day 9 of treatment, after which these improvements were lost [36]. This suggests that AuNPs may provide a therapeutic window of vascular normalisation, in which chemotherapy and radiotherapy could be more effectively utilised.

Of note, only 0.7% of systemically administered nanoparticles reach their intended site of the solid tumour [90]. Furthermore, the “passive targeting” approaches afforded by the EPR effect does not appear to be meaningfully replicated clinically, likely attributed to the considerable heterogeneity of the TME and metabolic differences of pre-clinical tumour models [91]. Therefore, tumour-targeted approaches are required to increase specificity. Integrins have become attractive anti-angiogenic targets given their role in tumour vascularisation [92]. In models of normal angiogenesis, liposome NPs functionalised with the αvβ3-integrin targeting peptide Arg-Gly-Asp (RGD) encapsulating doxorubicin exhibited potent antiangiogenic properties in vivo, inhibiting angiogenesis by up to 70% compared to controls [93]. In pre-clinical models, the co-functionalisation of AuNPs with RGD induced specific vascular damage in pancreatic tumour xenografts when coupled with image-guided radiation therapy [37]. Furthermore, RGD-functionalised AuNPs have also been shown to reduce MDA-MB-231 breast cancer cell invasiveness following radiotherapy in vitro [38]. Functionalisation of nanoparticles with other integrin targeting peptides such as the αvβ1-targeted ATN-161, which has been shown to reduce tumour microvessels by almost 50% in pre-clinical murine models of colon cancer when combined with 5-fluoroucail infusion, may improve vascular targeting and TME anti-angiogenic strategies [94].

### 2.4. Immunosuppression

A critical event in malignant tumour progression is the ability for tumour cells to acquire immunosuppression. Tumour hypoxia has been reported to promote an immunosuppressive microenvironment by recruiting regulatory T cells (Tregs), myeloid-derived suppressor cells (MDSCs) and tumour associated macrophages (TAMs) [95,96]. Tregs are an important stromal cell population that support tumour progression by immune evasion. Tumour hypoxia has been implicated in promoting the generation and the recruitment of Tregs via the production of TGF-β and chemokine ligand 28 (CCL28) (Figure 2). Treg recruitment and CCL28 expression were shown to be significantly upregulated under hypoxic conditions. In a xenograft model of liver cancer, CCL28 upregulation promoted tumour growth and Treg recruitment in vivo in a HIF-1α dependent manner. While knocking down of CCL28 could reverse hypoxia-induced recruitment, overexpression or knockdown of CCL28 did not pose any effect on colony formation or cell proliferation, indicating that CCL-28 most likely exerts its oncogenic role in a non-cell autonomous manner by recruiting Tregs [97].

Severe hypoxia has been observed in the colon of mice suffering from colitis-associated colon cancer (CAC), accompanied by reduced T cell CD4+ effector cell differentiation and the enhanced activity of suppressive Tregs. Furthermore, downregulation of pro-inflammatory cytokines (IL-2, IL-17, IFN-γ, and IL-9) and upregulation of the anti-inflammatory cytokine IL-10 was detected in CD4+ T cells stimulated under hypoxic conditions. Furthermore, the proportion of IFN-γ producing Th1 cells were significantly decreased under hypoxia. It is interesting to note that there is only a slight expression of PD-1 by CD4+Foxp-T cells in the colon of mice suffering from CAC compared to healthy control mice after stimulation under hypoxia. In contrast, significant upregulation of PD-1 in CD4+Foxp+ Tregs occurred following CAC hypoxic exposure. This indicates that hypoxia enhanced Tregs mediate immunosuppression rather than T-cell exhaustion dominating [98].

MDSCs represent another type of immune suppressor in the TME which has been proven to confer a negative impact on T-cell and NK cells. Hypoxia-promoted secretion of glioma-derived exosomes (GDEs) can be endocytosed by murine MDSCs. These hypoxia stimulated GDEs resulted in an enhanced ability to induce MDSCs activation and expansion compared to normoxic stimulated cells. This effect was mediated by targeting RAR-related orphan receptor alpha (RORA) and phosphatase and tensin homolog (PTEN) via miR-10a and miR-21 in GDEs (Figure 2) [99]. In addition, hypoxia has been reported to stimulate the migration of CD11b+Gr-1+ myeloid cells via the secretion of macrophage migration inhibitory factor (MIF) and interleukin-6 (IL-6) by head and neck squamous carcinoma (HNSCC). HIF-1α/2α dependent MIF and IL-6 regulate the chemotaxis, differentiation and pro-angiogenic function of CD11b+Gr-1+ myeloid cells. This is mediated through the binding of CD74/CXCR4, CD74/CXCR2 and subsequent activation of MAPK and PI3K/AKT signalling pathways. Knockdown of HIF-1α/2α fails to inhibit the migration of CD11b+Gr-1+ myeloid cells due to the compensatory effect of NF-κB under hypoxic condition, whereas dual blockade of HIF-1α/2α and NF-κB successfully inhibited this effect [100].

Hypoxia also serves as an important driver of MDSCs recruitment. In a model of hepatocellular carcinoma (HCC), HIF-1α upregulated the expression of chemokine (C-C motif) ligand 26 (CCL-26) in cancer cells, recruiting chemokine (C-X3-C motif) receptor 1 (CX3CR1) expressing-MDSCs to primary tumours and promoting HCC tumour growth. Furthermore, inhibition of CCL-26 by the HIF inhibitor digoxin or through the blockade of CX3CR1 using a neutralizing antibody suppressed MDSC recruitment and tumour growth [101]. Hypoxia can also directly promote the accumulation of MDSCs. Upregulation of ectonucleoside triphosphate diphohydrolase (ENTPD2) in HCC cell lines by hypoxia and HIF-1α, contributed to HCC tumour growth and MDSC accumulation. This effect was mediated by the prevention of MDSCs differentiation via the conversion of extracellular ATP to 5′-AMP by ENTPD2. As such, knockdown or inhibition of ENTP2 suppressed tumour growth, enhancing the efficacy of immune checkpoint inhibitors [102].

TAMs are one of the most abundant forms of immune cell populations within the TME, emerging as an important regulator in fostering malignancy, cancer progression and therapeutic resistance [103]. Tumour hypoxia has been proven to enhance TAM recruitment and infiltration via the hypoxia-induced secretion of chemokines (CCL-4, CCL-8,) and metabolites (lipoxygenase metabolites; Figure 2). Hypoxia and secreted macrophage soluble factors promote glioblastoma (GBM) invasiveness, though enhanced matrix metalloproteinase (MMP)-9 activity, promoting CCL4-CCR5 signalling between TAMs and U87 GBM tumour cells [104].

Macrophages can also undergo phenotypic changes and different forms of activation depending on the signal. Typically, classically activated macrophages are stimulated by T helper 1 (Th1) cytokines (TNF-α, IFN-γ) as well as microbial cell wall components. Indeed, the classical M1 macrophage phenotype is known to possess potential antibacterial and anti-inflammatory activity through the secretion of reactive oxygen species and nitrogen intermediates, thereby counteracting cancer progression. An alternative M2 macrophage immunosuppressive phenotype is stimulated by Th2 cytokines (IL-4, IL-13) and other cytokines, which are responsible for blocking Th1 response and promote angiogenesis and cell proliferating. Tumour hypoxia has been indicated to play a pivotal role in the phenotypical control of TAMs [105].

Hypoxia-induced extracellular vesicle (EV) miR-103a from lung cancer cells increased M2-type polarization, mediated by suppressed PTEN activity and the subsequent activation of the PI3/AKT and STAT pathway. Inhibition of miR-103a led to a decrease in hypoxia-induced M2-type polarization, while macrophages treated with EV miR-103a further enhanced cancer progression and tumour angiogenesis [106]. In addition, hypoxic-conditioned medium can push macrophages towards an M2 phenotype, mediated through the upregulation of neuropilin-1 (Nrp-1). Inhibition of Nrp-1 expression with siRNA can reduce the recruitment of macrophages and partially reversed the effect of hypoxia on the induction of the M2 phenotype [107].

Tumour derived exosomes enriched in immunosuppressive proteins including the chemokines/chemoattractant (CSF-1), monocyte chemoattractant protein-1/C-C chemokine 2 (MCP-1/CCL-2), and TGF-β, are also enhanced during hypoxia, leading to the macrophage recruitment and M2-like polarization both in vitro and in vivo [108]. Furthermore, hypoxia-induced exosomes can enhance oxidative phosphorylation in bone marrow. This occurs via the transfer of let-7a miRNA and subsequent suppression of insulin-Akt-mTOR pathway, resulting in the metabolic reprogramming of infiltrating monocytic macrophages. Hypoxia also inhibits T-cell anti-tumour functions through the accumulation of extracellular adenosine, induced through the increased expression of ectonucleotidase CD73 and CD39, both of which are products of HIF target genes [109,110]. Accumulated adenosine in the TME acts as a negative regulator of the anti-tumour T cell response, in which the binding of adenosine to A2AR triggers T-cell apoptosis, contributing to tumour immune evasion [111].

Upregulation of negative immune checkpoint molecule of programmed death ligand (PD-L1) in tumour cells, macrophages and dendritic cells under hypoxia, can initiate the interactions between PD-L1 and cell surface checkpoint receptor programmed cell death-1 (PD-1) expressed on effector T cells, resulting in the increased apoptosis of cytotoxic T lymphocytes (CTLs) and subsequent downregulation of T-cell antitumour reactivity. Increased PD-L1 and HIF target genes (CAIX and GLUT1) expression have been observed in a renal cell carcinoma (RCC) model possessing a VHL mutation. Furthermore, there is almost no PD-L1 expression in the presence of pVHL or absence of HIF-2α, indicating that PD-L1 expression is specifically regulated by the pVHL/HIF-2α axis in RCC [112]. Hypoxia exposure of DU145 and MDA-MB-231 cells led to the upregulation of PD-L1 expression in a HIF-1α dependent manner, where HIF-1α suppression led to a reduction in PD-L1 mRNA and cell surface protein in human prostate and murine melanoma cells. Furthermore, hypoxia induced resistance to CTL-mediated lysis in B16-OVA cells, which was abolished following a knockdown either HIF-1α or PD-L1 [113].

#### Reprogramming the Immunosuppressive TME with Nanotherapeutics

Immunotherapy has revolutionised cancer treatment. Immune checkpoint inhibitors, including those to PD-1, PD-L1 and CTL antigen 4 (CTLA-4), have now been approved for a number of cancers [114]. However, tumour immune evasion represents a major hurdle for the success of these therapeutics, which is potentiated during hypoxia as detailed above. Targeted approaches which temporally or spatially control the hypoxia-induced immune responses within the TME are essential, given that off-target and adverse effects may occur following manipulation of the immune system [115]. The use of nanotherapeutics as drug delivery approaches are now being considered to specifically modulate the immunosuppressive microenvironment of the tumour while sparing systemic immune modulation to induce an anti-tumour immune response [116].

Given their high plasticity, reprogramming or repolarisation of TAMs from an immunosuppressive M2-like phenotype to an anti-tumourigenic M1-like phenotype is an attractive approach. Furthermore, as these cells readily internalise particles, nanoparticles may be used to deliver agents directly to these cells. β-cyclodextrin nanoparticles loaded with the toll-like receptor (TLR) 7 and 8 agonist R848 were selectively delivered to TAMs in vivo, altering the TME phenotype to that of an M1 (Figure 1). Furthermore, when combined with the immune checkpoint inhibitor anti-PD-1, NPs significantly improved immunotherapy response rates, with complete tumour regression observed in almost 30% of CRC-bearing mice [39]. In another study, TAM reprogramming from an M2 to an M1 phenotype was also performed using baicalin-loaded PLGA nanoparticles containing a TLR 9 agonist and an antigenic peptide (HgP) to activate immune cells and promote anti-tumour immunity. NPs were further entrapped in a galactose-modified erythrocyte coating to increase biocompatibility and TAM targeting, the macrophage galactose-type lectin (MGL; CD301) receptor is expressed on myeloid antigen-presenting cells including macrophages. These biomimetic NPs also suppressed melanoma growth in vivo and increased the infiltration of CD8+ T cells into the TME [40].

Targeting the MDSC compartment may be another approach for improving immune tolerance within the TME. As mentioned above, PI3K signalling is crucial in the functioning of myeloid suppressor cells in response to chemokines, particularly the PI3K-γ isoform. PLGA NPs have been developed to incorporate the PI3K-γ inhibitor IPI-549, and co-functionalised to target the TME with aminoethyl anisamide (AEAA), a ligand for sigma-1 receptor which is overexpressed in a number of tumour types including pancreatic adenocarcinoma. IPI-549 loaded NPs significantly reduced both MDSC and tumour associated B cell proportions in KPC pancreatic tumour-bearing mice while also inhibiting tumour growth in comparison to free-IPI-549 [41]. An alternative approach, to repolarise MDSCs away from an immunosuppressive phenotype, has also been demonstrated in a glioma model in vivo. When combined with radiotherapy, magnetic zinc-doped iron oxide nanoparticles modified with polyethylenimine had the potential to reprogram MDSCs in the TME following intratumoural injection [42].

Lyp-1, a Nrp-1-binding peptide, homes to lymphatics, TAMs and tumour cells, particularly those within hypoxic regions of the tumour [117]. Lyp-1 conjugation has been shown to increase cellular uptake of PEG-PLGA (Poly (lactic-co-glycolic acid)) nanoparticles in lymphatic metastatic tumours in vivo [118]. Of particular interest, Lyp-1 may be used to target Nrp-1 expressing Tregs. Nanoparticles modified with the more potent truncated tLyp-1 peptide reduced immunosuppressive Tregs in the TME of murine B16/BL6 melanoma tumours in vivo, while activating intratumoural CD8+ T cells when combined with an anti-CTLA-4 immunotherapeutic (Figure 1). This approach also enhanced tumour inhibition and survival [43]. Limited infiltration and activation of CTLs, through Treg-mediated suppression, is another characteristic of the immunosuppressive TME. Recently, TME-activated nanoparticles conjugated with antibodies against PD-L1 have been co-loaded with the photosensitiser indocyanine green (ICG). Photodynamic therapy (PDT) combining ICG treatment with near-infrared (NIR) irradiation induced the generation of ROS, promoting intratumoral CTL infiltration. Furthermore, this nanoparticle and NIR combination therapy suppressed tumour growth and lung metastasis in the 4T1 murine mammary cancer model [44]. Interestingly, αvβ3-integrin (discussed in Section 2.3) has been shown to be a regulator of PD-L1, with αvβ3-integrin depleted tumour cells exhibiting reduced PD-L1 expression and increased CD8+ T cell infiltration in vivo. Furthermore, αvβ3-integrin blockade could prime tumours to anti-PD-1 therapy [119]. Therefore, effective functionalisation of nanoparticles to target and subsequently manipulate immune signalling or cells of the TME including Tregs and TAMs, may overcome the hypoxia-driven immunosuppression and also allow greater sensitising to immunotherapeutic strategies.

### 2.5. Metabolic Reprogramming

Molecular oxygen is a critical component of mitochondrial ATP production. However, tumour cells often forgo oxidative phosphorylation (OXPHOS) in the mitochondria in favour of increased glycolysis, even in the presence of oxygen. This aerobic glycolysis phenomenon in tumour cells is often known as the “Warburg effect”, following observations of Otto Warburg in the early twentieth century [120,121]. This metabolic transformation of cells can be enhanced by tumour hypoxia. HIF-1α has been reported to induce multiple changes in gene expression that mediate the switch from OXPHOS to glycolytic metabolism. These include the upregulation of glucose transporter-1 (Glu-1) and key glycolytic enzymes, such as lactate dehydrogenase A (LDHA), phosphoglycerate kinase 1 (PGK-1) and the hexokinase family of proteins (HK-1 and HK-II), resulting in enhanced glycolytic flux in order to meet cellular demands (extensively reviewed in [66]). Interestingly, bone marrow adipocytes have been shown to promote the Warburg effect in metastatic prostate cancer cells [122], which could be reversed following knockdown of HIF-1α. Furthermore, inhibition of HIF-1α hydroxylation and degradation via EV transmission of HIF-1α stabilising long noncoding RNA (HISLA) from TAMs has also been shown to enhance aerobic glycolysis and apoptotic resistance of breast cancer cells [123].

In addition to increasing the glycolytic activity, hypoxia can also suppress the production of mitochondrial ROS by uncoupling glycolysis and OXPHOS via the upregulation of pyruvate dehydrogenase kinase-1 (PDK-1). Hypoxia-induced PDK-1 expression can block the conversion of pyruvate to acetyl-CoA, hereby preventing ATP production via the TCA cycle, attenuating ROS production, which in turn protects cancer cells from hypoxia-induced apoptosis [124]. Hypoxia-induced expression of PDK-1 has also been shown in pancreatic cells to reduce pyruvate dehydrogenase (PDH) activity through phosphorylation of the E1α subunit at serine 232 [125]. More importantly, a clinical cohort of head and neck squamous cell carcinoma biopsies has shown that patients with phosphorylated E1α or expression of PDK-1 tend to experience a poorer clinical outcome.

Hypoxia attenuation of metabolism may also occur through modulating the function of the electron transport chain via the downregulation of cytochrome-c oxidase (COX, complex IV). HIF-1α have been shown to activate the transcription of genes encoding COX4-2 and the mitochondrial protease LON, leading to the degradation of the COX4-1 subunit, aiding in better adaption to hypoxia with reduced ROS production [126]. Hypoxia has also been shown to modulate monocaroboxylate transport expression (MCT), a group of transmembrane proteins responsible for the regulation of lactate metabolism. Hypoxia-induced MCT-1 plasma membrane expression, both in vitro and in vivo, can promote the glycolytic phenotype of glioblastomas. In addition, inhibition of MCT-1 significantly reduced lactate production, cell proliferation and invasion [127]. Furthermore, HIF-1α knockdown in the SW48 CRC cell line has also been shown to reduce MCT-4 expression in vitro [128], indicating that MCTs could be potentially acted on as therapeutic targets.

#### Nanotherapeutics to Target or Overcome Metabolic Reprogramming

The metabolic transformation of tumour cells to favour glycolysis over OXPHOS represents a promising therapeutic target that may be exploited by nanoparticles. Among the hexokinase family of proteins, HK-2, which catalyses the phosphorylation of glucose, is frequently overexpressed in tumour cells [129]. As a result, inhibitors to HK-2, including 3-bromopyruvate (3-BP), have been utilised to inhibit glycolysis and subsequently induce cell growth arrest [130]. However, the potential for off-target effects means that clinical applications have been limited. AuNPs targeted to the mitochondria and functionalised with 3-BP were able to suppress tumour cell glycolysis, in addition to reducing mitochondrial OXPHOS in PC3 and DU145 prostate cancer cells in vitro (Figure 1). Furthermore, the anti-cancer potential of these AuNPs was potentiated in tumour cells when combined with laser irradiation in comparison to normal hMSC cells, which displayed no significant toxicity following treatment [45]. MCT-4 is upregulated due to the high rate of glycolysis, which is responsible for lactate/H^+^ across the cell membrane and the induction of an acidic tumour microenvironment [131]. Targeting MCT-4 represents another promising approach for modulating glycolysis. For example, Liu et al. (2018) loaded the amorphous iron oxide NPs with siRNA targeting MCT-4 for the treatment of prostate cancer [132]; the author found that significant suppression of MCT-4 expression and enhanced Fenton-like reaction-induced oxidative damages were seen both in vitro and in vivo, leading to a significant inhibition in tumour growth.

An alternative approach to overcome hypoxia-driven metabolic reprogramming within the TME is to alleviate tumour hypoxia by reducing the oxygen consumption (OC) within cells. To this end, a limited number of therapeutics have been identified with the potential to reduce the OC in tumour cells including the anti-malarial atovaquone and the anti-diabetic metformin. Atovaquone has been shown to reduce the OC by more than 80% in a number of tumour cells in vitro, and could abolish hypoxia in xenograft models of head and neck cancer and colon cancer after seven days of treatment, by inhibiting mitochondrial complex III. This was also associated with an improved radiation tumour growth response in vivo (growth delay of 13.2 days between control and atovaquone irradiation groups) [133]. NPs formulations containing atovaquone have previously been developed as long-acting chemoprophylaxis for malaria in pre-clinical models [134], and to improve the bioavailability of the drug [135]. More recently, co-functionalised NPs have been developed encapsulating atovaquone with either the photosensitiser veterporfin or ICG to improve PDT responses in 4T1 mammary tumour-bearing and HeLa cervical adenocarcinoma-bearing animals in vivo, by increasing intratumoural oxygenation resulting in greater anti-tumour effects [46,47].

Conversely, the anti-diabetic metformin has been shown to reduce OC by inhibiting complex I in the mitochondrial electron transport chain, leading to improvements in tumour oxygenation and radiation responses in models of colon cancer in vivo [136]. PEG-PCL (poly(ε-caprolactone)) liposome NPs containing metformin and a photosensitizer (IR780) have also been shown to decrease endogenous OC in gastric cancer cells in vitro and increase ROS generation. In vivo, these co-functionalised NPs could overcome hypoxia and improve PDT and photothermal therapy (PPT) to attenuate tumour growth [48]. Tungsten oxide NPs including W_18_O_49_ have been used as effective PDT and PPT agents due to their ability to generate ROS and produce heat when combined with NIR laser irradiation [137]. However, this effect may be limited by hypoxia, as such the development of platelet membrane NPs co-loaded with metformin and W_18_O_49_ have been shown to improve responses to PDT and PPT in vitro by reducing the OC in tumour cells, and significantly inhibiting tumour growth and increasing TUNEL staining (a measure of tumour apoptosis) in Raji-lymphoma xenografts [49].

## 3. Conclusions and Future Perspectives

Tumour hypoxia is a critical feature of the TME, contributing to disease progression and resistance to chemo and radiotherapy. The response to tumour hypoxia is mainly driven by oxygen-dependent HIFs that enable tumour progression. However, a complete understanding of the mechanisms driving the response to hypoxia within the TME remains elusive. Hypoxia has effects not only on tumour cells and on the maintenance of CSCs, but also on those cells of the surrounding stroma, driving angiogenesis, malignant progression, immunosuppression and aiding in metabolic reprogramming (Figure 2). Despite the fact that nanotherapeutics hold real potential for targeting these physiological and pathological responses to hypoxia in the TME (summarised in Table 1), most of these hypoxic based nanotherapeutics remain at a preliminary stage of development.

Significant barriers to the translation of NPs include sufficient tumour penetration, stability and potential systemic toxicity. However, meaningful achievements have been made to improve nanomedicine delivery and retention in solid tumours through smart nanoparticle design and TME modification. Development of self-recognition biomimetic nanodelivery systems have shown great potential in increasing the circulation and biocompatibility of nanoparticles within the host organism; this includes coating NPs with an erythrocyte or cancer cell membrane. Alternatively, enhanced tumour penetration could be achieved through the use of circulating monocytes or macrophages [138]. Tumour pre-treatments with radiation therapy or mild hyperthermia represent another promising approach for improving nanoparticle deposition and intratumoural distribution [139]. Furthermore, given that the EPR effect has been shown to be not recapitulated clinically, approaches that improve the distribution of NPs into hypoxic regions of the TME are required. Vascular normalisation or ECM modification is one approach that has been shown to increase the intratumoural accumulation and distribution of nanomedicine in preclinical models, which may also improve tissue oxygenation alleviating hypoxia within the TME [140,141,142]. However, alternative approaches to improve tissue penetration may be afforded by direct intratumoural administration of NPs.

Future research efforts and clinical translation of nanotherapeutics will require a detailed understanding of the molecular nature of the hypoxic TME to optimise treatment combinations. This approach will undoubtedly facilitate the development of more promising nanotherapeutic platforms for the future treatment of hypoxic tumours that are not only targeted towards tumour cells, but have dual-targeting effects on cells of the TME in addition to augmenting intratumoural oxygenation.

## Figures and Tables

**Table 1:** Summary of nanotherapeutic strategies to overcome hypoxia-mediated progression.

Nanoparticle Formulation	Drug/Therapeutic	Targeting Moiety	Target	Indications and Measured Benefit	Ref.
***2.1 Enrichment and propagation of cancer stem cells***					
PLGA	Salinomycin	CD133 aptamer	CD133 receptor	Selectively kill CD133^+^ osteosarcoma cells et and in vivo and reduce tumoursphere formation and the percentage of Sao-2 CD133^+^ cells	[15]
PLGA-PEG	Salinomycin	CD133 Antibody	CD133 receptor	Reduction in the percentage of CD133^+^ ovarian cancer cells. 2.5- fold decrease in PA-1tumor sphere number compared to the saline control	[16]
Lipid polymers	Salinomycin	CD133 and EGFR aptamer	CD133 receptor EGFR	Targeting both osteosarcoma CSCs and cancer cells with high specificity, 90% decrease in tumour volume	[17]
Lipid polymers	ATRA	CD133 aptamer	CD133 receptor	Osteosarcoma tumour volume inhibitory rate for the ATRA-PLNP-CD133 treated group was 81.1%	[18]
Hyaluronic acid and styrene-maleic acid Nano micelle	Curcumin	Hyaluronic acid	CD44 receptor	Marked inhibition of NF-κB signalling and significant reduction in CD44+ expression cells in pancreatic cancer cells	[19]
Pluronic f127	Doxorubicin	Chitosan	CD44 receptor	Increased the toxicity of doxorubicin (Dox)by six times compared to free Dox in eliminating CD44+ CSC-like cells in MCF-7 breast cancer (BCa) cells.	[20]
Liposome	Salinomycin Doxorubicin			A significant decrease in liver cancer stem cells in vivo (HepG2, HepG2-TS cells)	[21]
PLGA	Salinomycin Paclitaxel	Hyaluronic acid	CD44 receptor	A significant reduction in CD44+ cells in Breast cancer, MCF-7 and MDA-MB-231 cells	[22]
PEG-PLA	Doxorubicin ATRA			Induced differentiation of CSCs and sensitized cells toward DOX treatment. Combinatory treatment significantly reduces MDA-MB-231 tumour growth in vivo.	[23]
Mesoporous silica	Cisplatin, 5-fluoroucail, paclitaxel	siRNA	ABCG2	Downregulation of ABCG2 significantly enhanced the drug-induced apoptosis and inhibited Hep-2 (laryngeal) tumour growth in vivo.	[24]
PLGA	Paclitaxel	Wedelolactone	SOX-2, ABCG2	Wedelolactone treatment sensitizes MDA-MB-231 BCa cells to the effects of paclitaxel and significantly reduced the ALDH+ breast cancer CSCs and suppressed the tumour growth	[25]
Silica	γ-secretase inhibitor		Notch signalling	Breast cancer, MDA-MB-231 cells. Reduce ALDH side population in CAM model and suppressed tumor growth in vivo	[26]
***2.2 Invasion and Metastasis***					
AuNPs			MAPK signalling	Inhibited the proliferation of SKOV3 (ovarian) cancer cells and delayed the tumoral and metastases growth by reversing EMT and inhibition of MAPK signalling	[27]
PEI coated SPIONs			Src kinase, miR-21, MMP2	Reduced the invadosome intensity and decreased the ability of Pan02 (pancreatic cancer) cells to invade through basement membrane.	[28]
FA-PEG-PEI-SPIONs		miR-125b-5p	JAK-STAT, Wnt/β-Catenin	Inhibited the invasion, migration, and growth of HCC HUH7 and HCCLM3 cells	[29]
Zinc arsenite			SHP-1/JAK-STAT	Inhibit tumour growth of HCC xenografts by 2.2-fold and metastasis by 3.5-fold as compare free arsenic trioxide-based NPs	[30]
PEG-AuNPs			PI3/AKT	Supressed tumour growth and decrease sphere formation of glioblastoma and lung adenocarcinoma A549 cells	[31]
Hyaluronic acid conjugated NPs	cisplatin	siRNA	Snail, Twist	Knockdown Twist and reversed chemoresistance to reduce tumour growth and metastasis of Ovarian cancer, F2 and Ovacar 8 cells in vivo	[32]
Amphiphilic polymers	paclitaxel	siRNA	Snail, Twist	Inhibited tumour growth and metastasis of 4T1 tumours in vivo simultaneously	[33]
***2.3 Angiogenesis***					
AuNPs			VEGF, bFGF	inhibited endothelial /fibroblast cell proliferation & angiogenesis in an ovarian cancer model in vivo	[34]
AuNPs			EMT, MMP-2	Facilitated tumour vasculature normalization, increased blood perfusion and alleviate tumour hypoxia in a model of lung cancer (B16F10) in vivo	[35]
AuNPs			Anterior gradient 2 (AGR2)	Reduced vessel density, tumour volume and increased the pericyte coverage in metastatic CRC model (SW620) in vivo	[36]
AuNPs		RGD	αvβ3	Induced tumour vascular disruption and improved the therapeutic outcome of radiotherapy of Panc-1 pancreatic tumours in vivo	[37]
AuNPs		RGD	αvβ3	Reduced breast cancer (MDA-MB-231) cell viability and increased DNA damage compared to radiation alone in vitro.	[38]
***2.4 Immunosuppression***					
β-cyclodextrin	TLR7/8 agonist (R848)	Cyclodextrin	Engulfed by TAMs	Remodelled TME from M2 to M1 phenotype. Improved anti-PD-1 response rates in murine colon cancer models	[39]
PLGA	TRL9 agonist	Galactose	MGL—TAMs	Reprogrammed TAMs from M2-M1, suppressed melanoma tumour growth and increased CTL infiltration in vivo	[40]
PLGA	PI3K-γ inhibitor (IPI-549)	AEAA	Sigma-1 receptor—TME	Reduced MDSC proportion and decreased tumour growth in pancreatic tumour model in vivo	[41]
Magnetic zinc-doped iron oxide			MDSC	Repolarise MDSCs from immunosuppressive to pro-inflammatory when combined with Rad.	[42]
PLGA	Tyrosine kinase inhibitor (Imatinib)	Lyp-1	Nrp-1—Tregs	Enhanced tumour inhibition and survival of murine melanoma tumours when combined with immune checkpoint inhibitor	[43]
PLGA	Anti-PD-L1 & ICG	MMP-2 sensitive property	MMP-2—TME	Increased CTL tumour infiltration. Suppressed tumour growth and lung metastasis in 4T1 breast cancer model	[44]
***2.5 Metabolic reprogramming***					
AuNPs	3-BPP		HK2 (mitochondria)	Suppressed tumour cell glycolysis and OXPHOS in prostate cells in vitro	[45]
PLGA	Atovaquone + Veterporfin		Complex III (mitochondria)	Improved intratumoural oxygenation and anti-tumour response to PDT in 4T1 tumour bearing mice	[46]
Gelatin	Atovaquone + ICG		Complex III (mitochondria)	Improved intratumoural oxygenation and anti-tumour response to PDT in HeLa xenografts	[47]
PEG-PCL	Metformin + IR780		Complex I (mitochondria)	Decreased endogenous oxygen consumption in gastric cancer cells in vitro. Improved PDT and PTT in vivo	[48]
Tungsten oxide (W_18_O_49_)	Metformin		Complex I (mitochondria)	Lowered OCR and inhibit tumour growth in Raji-lymphoma-bearing mice	[49]
